# Biological and Clinical Relevance of Genetic Alterations in Peripheral T-cell Lymphomas

**DOI:** 10.31662/jmaj.2024-0405

**Published:** 2025-03-07

**Authors:** Yuta Ito, Yasunori Kogure, Keisuke Kataoka

**Affiliations:** 1 Division of Molecular Oncology, National Cancer Center Research Institute, Tokyo, Japan; 2 Division of Clinical Oncology and Hematology, Department of Internal Medicine, The Jikei University School of Medicine, Tokyo, Japan; 3 Division of Hematology, Department of Medicine, Keio University School of Medicine, Tokyo, Japan

**Keywords:** Peripheral T-cell lymphoma, genetic alterations, clinical application, nodal T follicular helper cell lymphomas, anaplastic large cell lymphoma, adult T-cell leukemia/lymphoma, extranodal natural killer/T-cell lymphoma

## Abstract

Peripheral T-cell lymphoma (PTCL) is a heterogeneous group of mature T-cell neoplasms with different clinical, biological, and molecular features. These include PTCL, not otherwise specified, nodal T follicular helper cell lymphomas (nTFHLs), anaplastic large cell lymphoma (ALCL), extranodal natural killer (NK)/T-cell lymphoma (ENKTL), and adult T-cell leukemia/lymphoma (ATLL). Over the past decade, several genetic studies using targeted, whole-exome, and more recently whole-genome sequencing have identified numerous driver alterations in PTCLs. These alterations include mutations, copy number alterations, and structural variations (SVs) involving T-cell receptor/NF-κB (such as *PLCG1*, *VAV1*, and *CD28*) and JAK/STAT (*JAK3* and *STAT3*) pathway components, epigenetic regulators (*TET2*, *DNMT3A*, and *ARID1A*), immune-associated molecules (*HLA-A/B*, *CD58*, and *PD-L1*), and tumor suppressors (*TP53* and *CDKN2A*), which are shared among various PTCL subtypes. Conversely, subtype-specific alterations, such as *RHO*A G17V and *IDH2* R172 mutations in nTFHLs; *ALK* fusions in ALCL; *DDX3X* and *MSN* mutations in ENKTL; and *PRKCB*, *CIC,* and *CCR4* mutations in ATLL. Regarding the clinical relevance of genetic alterations, combining genetic information with clinical factors has been reported to improve prognostic stratification in several subtypes of PTCLs, such as ENKTL and ATLL. Additionally, several genetic alterations may have the potential to predict a response to a specific molecularly targeted agent, such as *ALK* fusions for ALK inhibitors, *PD-L1* SVs for immune checkpoint inhibitors (including anti-PD-1 antibodies), and mutations in epigenetic regulators for histone deacetylase inhibitors and hypomethylating agents. In this study, we summarize the current understanding of somatic alterations in various subtypes of PTCLs and highlight their clinical utility.

## Introduction

Peripheral T-cell lymphoma (PTCL) is a heterogeneous entity comprising mature T-cell neoplasms with different clinical, biological, and genetic characteristics ^(1), (2)^. According to the 5^th^ edition of the World Health Organization (WHO) Classification of Haematolymphoid Tumors (WHO-HAEM5) and the International Consensus Classification (ICC), this category includes PTCL, not otherwise specified (PTCL-NOS), nodal T follicular helper (TFH) cell lymphomas (nTFHLs), anaplastic large cell lymphoma (ALCL), extranodal natural killer (NK)/T-cell lymphoma (ENKTL), adult T-cell leukemia/lymphoma (ATLL), cutaneous T-cell lymphoma (CTCL, such as Sézary syndrome and mycosis fungoides), and others ^(1), (2)^.

Conventionally, most patients with PTCL are treated with anthracycline-containing regimens such as cyclophosphamide, doxorubicin, vincristine, and prednisolone (CHOP) or CHOP-like regimens ^(3)^. After achieving remission with chemotherapy, some patients undergo autologous stem cell transplantation as consolidation therapy, which may improve patient outcomes in certain subtypes of PTCL. In addition, allogeneic stem cell transplantation offers a potential cure for some patients, especially those with relapsed or refractory diseases, although its role in PTCL remains controversial. With respect to novel agents, the ECHELON-2 study demonstrated that the combination of brentuximab vedotin and cyclophosphamide, doxorubicin, and prednisolone (CHP) showed significantly superior progression-free survival for CD30-positive PTCL, particularly ALK-positive ALCL ^(4)^. Furthermore, epigenetic modifiers, including histone deacetylase (HDAC) inhibitors (such as romidepsin, belinostat, and chidamide [tucidinostat]) as well as hypomethylating agents (such as azacitidine), are effective against various subtypes of PTCL, particularly nTFHL, angioimmunoblastic type (nTFHL-AI, previously called as angioimmunoblastic T-cell lymphoma [AITL]) ^(3), (5)^. Moreover, subtype-specific treatment modalities have been developed for several subtypes of PTCL, particularly ALK-positive ALCL, ENKTL, and ATLL (described in detail later).

In patients receiving conventional treatment, prognosis varies among PTCL subtypes: ALK-positive ALCL has the best 5-year overall survival (OS) of >70%, whereas ATLL has the worst 5-year OS of <20% ^(6), (7)^. The prognosis of PTCL-NOS, nTFHL-AI, and ENKTL is intermediate between these. Historically, the International Prognostic Index (IPI), which was developed for aggressive non-Hodgkin lymphoma, has been widely used for prognostication and risk stratification in PTCLs ^(6)^. Subsequently, several prognostic indices, such as the Prognostic Index for T-cell Lymphoma (PIT), modified PIT, and International Peripheral T-cell Lymphoma Project (IPTCLP) score, have also been developed for PTCL-NOS and/or nTFHL-AI ^(8), (9), (10)^. More recently, several subtype-specific prognostic indices, such as the prognostic index for AITL (PIAI), AITL score, prognostic index of natural killer lymphoma (PINK), and prognostic index for acute- and lymphoma-type ATLL (ATL-PI), have been proposed (described in detail later) ^(11), (12), (13), (14)^.

## Nodal T Follicular Helper Cell Lymphomas

nTFHL (also known as follicular helper T-cell lymphoma in ICC) represents a group of mature T-cell neoplasms with immunophenotypic features and gene expression signatures of TFH cells ^(1), (2)^. TFH cells are a subset of effector T-helper cells that predominantly reside in secondary lymphoid follicles and are essential for germinal center formation, affinity maturation, and memory B cell development. nTFHL comprises three subtypes: nTFHL-AI, nTFHL follicular type (nTFHL-F), and nTFHL not otherwise specified (nTFHL-NOS). Neoplastic T cells exhibit a spectrum of TFH-associated immunophenotypic markers, such as PD-1, ICOS, CXCL13, CD10, BCL6, CXCR5, SAP, c-MAF, and CD200, with the first five being more commonly available in the diagnostic setting. nTFHL-AI is the prototype with well-defined morphological, immunophenotypic, and genetic profiles. This subtype is characterized by systemic disease and a polymorphous lymphoid infiltrate involving the lymph nodes, accompanied by prominent proliferation of endothelial venules and follicular dendritic cells.

**Figure 1.**
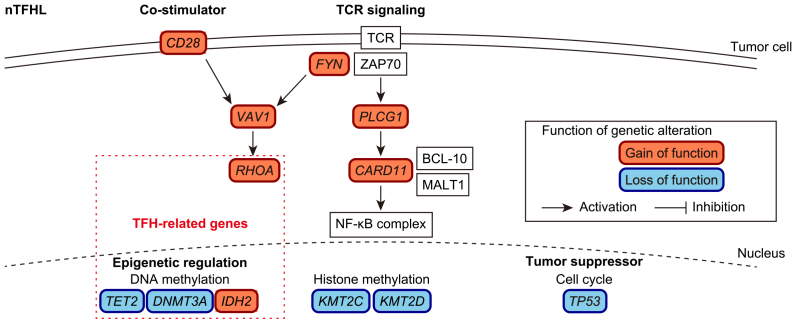
Frequent genetic alterations in nTFHLs are summarized according to their functionalities. Gain- and loss-of-function alterations are shown in orange and blue, respectively.

Recent genetic studies have shown the stepwise accumulation of somatic alterations during nTFHL-AI lymphomagenesis (Figure 1). In the early phase, hematopoietic stem cells acquire clonal hematopoiesis (CH)-associated mutations, such as *TET2* and *DNMT3A* mutations ^(15)^. A recent study demonstrated that CH-associated mutations promote the expansion of germinal center B cells, which in turn facilitates the development of T-cell lymphomas in an nTFHL-AI mouse model ^(16)^. Other genetic alterations occur later in lineage-committed cells that develop into T-cell lymphomas. Among these, mutation of* RHOA*, which encodes a small GTPase, has the highest frequency (observed in ~70% of nTFHL-AI cases), with p. G17V being the most prevalent ^(17), (18), (19)^. Neomorphic *IDH2* mutations, which encode an isocitrate dehydrogenase, are present in ~30% of cases ^(20)^. In mouse models, *Tet2* inactivation combined with *Rhoa* p. G17V or *Idh2* p. R172K mutant expression in CD4^+^ T cells induces T-cell lymphomas that recapitulate nTFHL-AI through deregulation of the transcriptomic and/or epigenetic profiles of TFH cells ^(21), (22)^. In addition to these genes, recurrent mutations are present in various epigenetic regulators (including *KMT2C* and *KMT2D*) ^(23)^. In addition, several genetic alterations enhance T-cell receptor (TCR) and co-stimulatory signaling, including mutations in *CD28*, *VAV1*, *PLCG1*, *CARD11*, and *FYN,* as well as *CTLA4*::*CD28* and *ICOS*::*CD28* fusions ^(24), (25)^. *TP53* is also affected in nTFHL-AI although its frequency is low (<5%) ^(26)^.

On the contrary, nTFHL-F and nTFHL-NOS, previously termed follicular T-cell lymphoma and PTCL with TFH phenotype, respectively, represent less-characterized nodal lymphomas that also express TFH molecules ^(1), (2)^. nTFHL-F exhibited a follicular growth pattern, whereas nTFHL-NOS lacked the characteristic histopathological features of nTFHL-AI or nTFHL-F. Due to their low frequency and recent recognition in the WHO and ICC classifications, genetic and clinical differences among the three nTFHL subtypes remain unclear.

In addition to IPI and PIT, several subtype-specific prognostic models (PIAI and AITL scores) have been proposed for nTFHL-AI. The PIAI comprises the following factors: older age (>60 years), poor performance status (≥2), extranodal sites >1, B symptoms, and low platelet count (<150 × 10^9^ /L). The AITL score is based on older age (≥60 years), poor performance status (>2), elevated C-reactive protein, and elevated β2-microglobulin. Regarding the prognostic impact of genetic alterations, to date, no association between prognosis and frequently observed driver mutations, such as those involving *RHOA*, *TET2*, *IDH2*, and *DNMT3A*, has been reported in nTFHL. Probably reflecting a high frequency of mutations involving epigenetic regulators, nTFHLs have been shown to demonstrate a high response rate to HDAC inhibitors (such as romidepsin, belinostat, and chidamide) and hypomethylating agents (such as azacitidine), although the predictive value of individual gene mutations has not been clearly established ^(27), (28), (29), (30), (31)^.

## PTCL, Not Otherwise Specified

PTCL-NOS is a heterogeneous category of nodal and extranodal T-cell lymphomas that do not meet the criteria for any specific PTCL subtypes and has been considered a “waste basket” category. Gene expression profiling studies have classified PTCL-NOS into two molecular subtypes based on the expression of two transcription factors, TBX21 and GATA3, which regulate T helper 1 (Th1) and Th2 differentiation, respectively ^(32)^. PTCL-GATA3 is associated with worse survival compared with PTCL-TBX21. Regarding genetic alterations, *TP53* and/or *CDKN2A* mutations and deletions are frequently observed and form a distinct molecular subtype in PTCL-NOS ^(33), (34), (35)^. This subtype is associated with PTCL-GATA3 and exhibits widespread genomic instability, which preferentially involves molecules associated with immune escape, such as *HLA-A*, *HLA-B*, and *CD58*. In addition, PTCL-NOS exhibits recurrent alterations in CH-associated genes (*DNMT3A* and *TET2*), epigenetic regulators (*KMT2C*,* KMT2D*,* SETD1B*,* SETD2*,* CREBBP*, *ARID1A*, and* ARID2*), and TCR signaling molecules (*PLCG1*, *CD28*, *CARD11*, *TNFAIP3*, and *PTPRC*) ^(33)^. Within the PTCL-TBX21 subgroup, *DNMT3A* mutation defines a unique subgroup associated with the cytotoxic T-cell phenotype and a worse prognosis ^(36)^. Conversely, there is a subset that lacks the driver alterations characteristic of PTCLs and shows a better prognosis ^(33)^. These observations suggest clinical and genetic heterogeneity in PTCL-NOS.

## Anaplastic Large Cell Lymphoma

ALCL involves the proliferation of predominantly large lymphoid cells with high CD30 expression and is divided into two groups based on ALK expression ^(1)^. ALK-positive ALCL generally has a favorable prognosis and harbors a defining structural variation (SV) that fuses the 3′ portion of *ALK* on chromosome 2p23 with the 5′ portion of a partner gene that functions as a promoter. Although more than 20 partner genes have been reported to be involved in *ALK* SVs, *NPM1*::*ALK* is the most common (>80% of cases), followed by *TPM3*::*ALK*. These SVs cause constitutive expression of the kinase function, triggering numerous cellular signaling pathways, especially the JAK/STAT pathway. Molecularly targeted agents, including small-molecule ALK inhibitors, have the potential to improve the prognosis of ALK-positive ALCL, particularly in patients with relapsed or refractory diseases ^(37)^.

Conversely, ALK-negative ALCL possesses recurrent gain-of-function mutations in *JAK1* and/or *STAT3*, as well as rare SVs involving tyrosine kinase genes, such as *ROS1* and *TYK2* ^(38)^. These genetic alterations also activate the JAK/STAT pathway, which may underlie the pathological and biological similarities between ALK-positive and ALK-negative ALCLs. SVs involving the *DUSP22* locus at 6p25.3 occur in approximately 20%-30% of cases ^(39), (40)^. In approximately 5% of cases, *TP63* SVs occur as an inversion of 3q28 involving the partner gene *TBL1XR1 and* are associated with worse outcomes.

## Extranodal NK/T-cell Lymphoma

ENKTL is an aggressive NK/T-cell neoplasm that predominantly occurs in Asian countries and frequently involves the upper aerodigestive tract, including the nasal cavity, nasopharynx, and paranasal sinuses ^(41), (42)^. ENKTL is characterized by angiodestructive, tissue necrosis, and cytotoxic phenotype and is strongly associated with Epstein-Barr virus (EBV). ENKTL generally exhibits a type II latency pattern characterized by latent membrane proteins (LMPs) 1 and 2 and EBV nuclear antigen 1 (EBNA1) ^(43)^. Although their precise oncogenic roles have not been well investigated in the ENKTL pathogenesis, these viral products, particularly LMP1, transcriptionally upregulate MYC expression and activate the NF-κB and JAK/STAT pathways, which can contribute to NK/T-cell transformation, survival, and proliferation.

**Figure 2.**
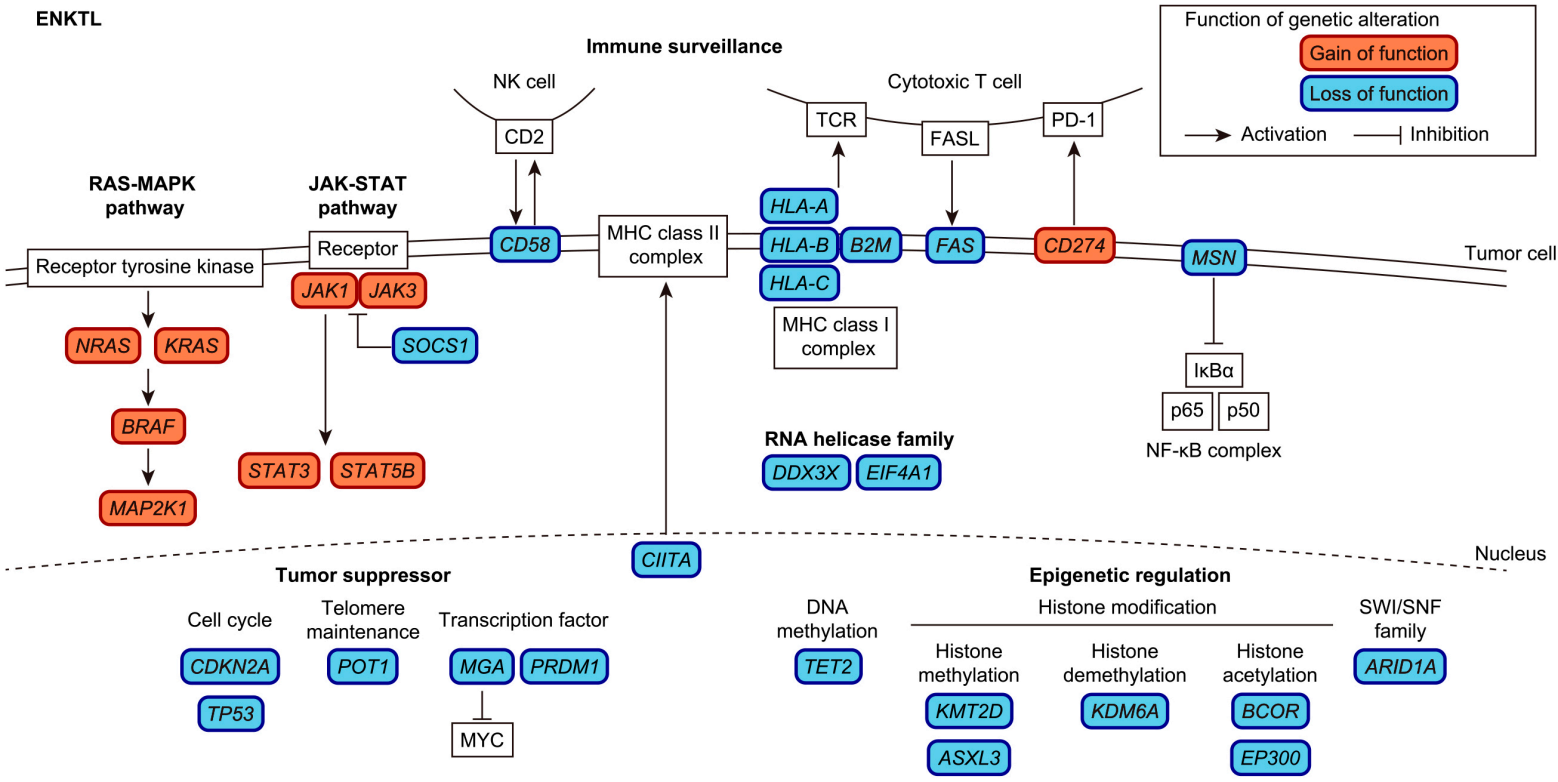
Frequent genetic alterations in ENKTL are summarized according to their functionalities. Gain-of-function and loss-of-function alterations are shown in orange and blue, respectively.

In addition to EBV infection, acquired somatic alterations are considered essential for the development and progression of ENKTL. Several recent genetic studies have revealed the entire landscape of genetic alterations in ENKTL and have identified recurrent somatic alterations (Figure 2) ^(44), (45), (46), (47), (48)^. The most frequent genetic alterations in ENKTL are SV and amplifications involving *PD-L1* (also known as *CD274*) ^(48), (49)^. These SVs include a variety of SV types (deletion, inversion, tandem duplication, and translocation), most of which result in 3′-untranslated region (UTR) truncations, causing constitutive activation of PD-L1 ^(49), (50)^. Approximately half of the ENKTL cases possessed somatic alterations in immune-associated molecules, including MHC class I components (*HLA-A/B/C* and *B2M*), an MHC class II transactivator (*CIITA*), and molecules involved in cell adhesion (*CD58*) and death signaling (*FAS*), together with *PD-L1* ^(44), (45), (46), (47), (48)^. The second most altered pathway is epigenetic regulation, consisting of molecules involved in histone modification (*BCOR*, *KMT2D*, *ASXL3*, *KDM6A*, and *EP300*), chromatin remodeling (*ARID1A*), and DNA methylation (*TET2*). Other commonly altered pathways include tumor suppressors (*TP53*, *CDKN2A*, and *POT1*), transcription factors (*PRDM1* and *MGA*), RNA helicase family (*DDX3X* and* EIF4A1*), the JAK/STAT pathway (*JAK1*, *JAK3*, *STAT3*, *STAT5B*, and *SOCS1*), and the RAS/MAPK pathway (*KRAS*, *NRAS*, *BRAF*, and *MAP2K1*).

Among arm-level copy number alterations (CNAs), chromosome X deletions are the most prevalent and are exclusively observed in females. In addition, ENKTL cases harbor frequent disruptions of X-linked driver genes (*MSN*, *BCOR*, *DDX3X*, and *KDM6A*), which are more common in males and females with chromosome X losses ^(48)^. Among the X-linked drivers,* MSN* was the most frequently altered factor, and disruption of *MSN* was reported to promote cell proliferation, activate the NF-κB pathway, and confer higher sensitivity to NF-κB inhibition. These observations suggest that driver genes on chromosome X are coordinately involved in ENKTL pathogenesis and may explain male predominance in ENKTL.

In addition to genetic alterations involving the host genome, frequent deletions within the EBV genome have been reported in EBV-associated lymphoid neoplasms, including ENKTL. Among them, the most frequent are deletions affecting *Bam*HI-A rightward transcript (BART) microRNA clusters 1 and 2 ^(51), (52)^ which are shown to reactivate the lytic cycle through the upregulation of two immediate early genes, *BZLF1* and *BRLF1*, thus contributing to lymphomagenesis ^(53)^. The second most common is deletions at the origin of replication (oriP), which consists of two essential components: the family of repeats and the dyad symmetry sequence ^(54)^. These components contain multiple binding sites for EBV nuclear antigen-1 (EBNA-1), which facilitates the replication and stable maintenance of EBV plasmids. Therefore, BART and oriP deletions appear to be involved in the development and progression of ENKTL through different molecular mechanisms.

Although the clinical outcome of ENKTL has substantially improved as a result of new treatment strategies with L-asparaginase-based chemotherapies and the upfront use of concurrent chemoradiotherapy, the prognosis of patients with advanced disease remains poor, with a 5-year OS rate of 40% ^(41), (42)^. Clinical prognostic models have been widely used, including PINK, which is based on four risk factors: older age (>60 years), advanced disease (stage III or IV), distant lymph node involvement, and non-nasal type, or PINK in combination with peripheral blood EBV DNA (PINK-E) ^(14)^. In addition, new molecular classifications have recently been proposed. For example, gene expression profiling showed that ENKTL cases can be divided into three transcriptomic subtypes: tumor suppressor (*TP53*) / immune modulator (JAK/STAT pathway and PD-L1) (TSIM), MGA-BRDT (MB), and HDAC9-EP300-ARID1A (HEA) subtypes ^(47)^. These subtypes differed significantly with respect to cell of origin, EBV gene expression, transcriptional signatures, and responses to L-asparaginase-based regimens and targeted therapy. In addition, our genetic study showed that genomic alterations can also classify ENKTL cases into two genetic subtypes ^(48)^. In this classification, group 1 is characterized by a larger number of driver alterations and is enriched with alterations involving tumor suppressors (*CDKN2A* and *TP53*) and gain-of-function mutations of the JAK/STAT (*JAK3* and *STAT5B*) and RAS/MAPK (*NRAS*) pathway molecules. In contrast, *PD-L1* SVs, amplifications, mutations, and deletions affecting *MSN* and *ARID1A* were more frequently observed in group 2. Importantly, group 1 had a worse prognosis than group 2, independent of the PINK score. In addition to prognosis, genetic alterations can predict response to molecularly targeted agents. In particular, it has been reported that patients harboring *PD-L1* SVs show superior outcomes following PD-1 blockade therapy, suggesting its utility as a predictive biomarker for immune checkpoint inhibitors ^(55)^.

## Adult T-cell Leukemia/Lymphoma

**Figure 3.**
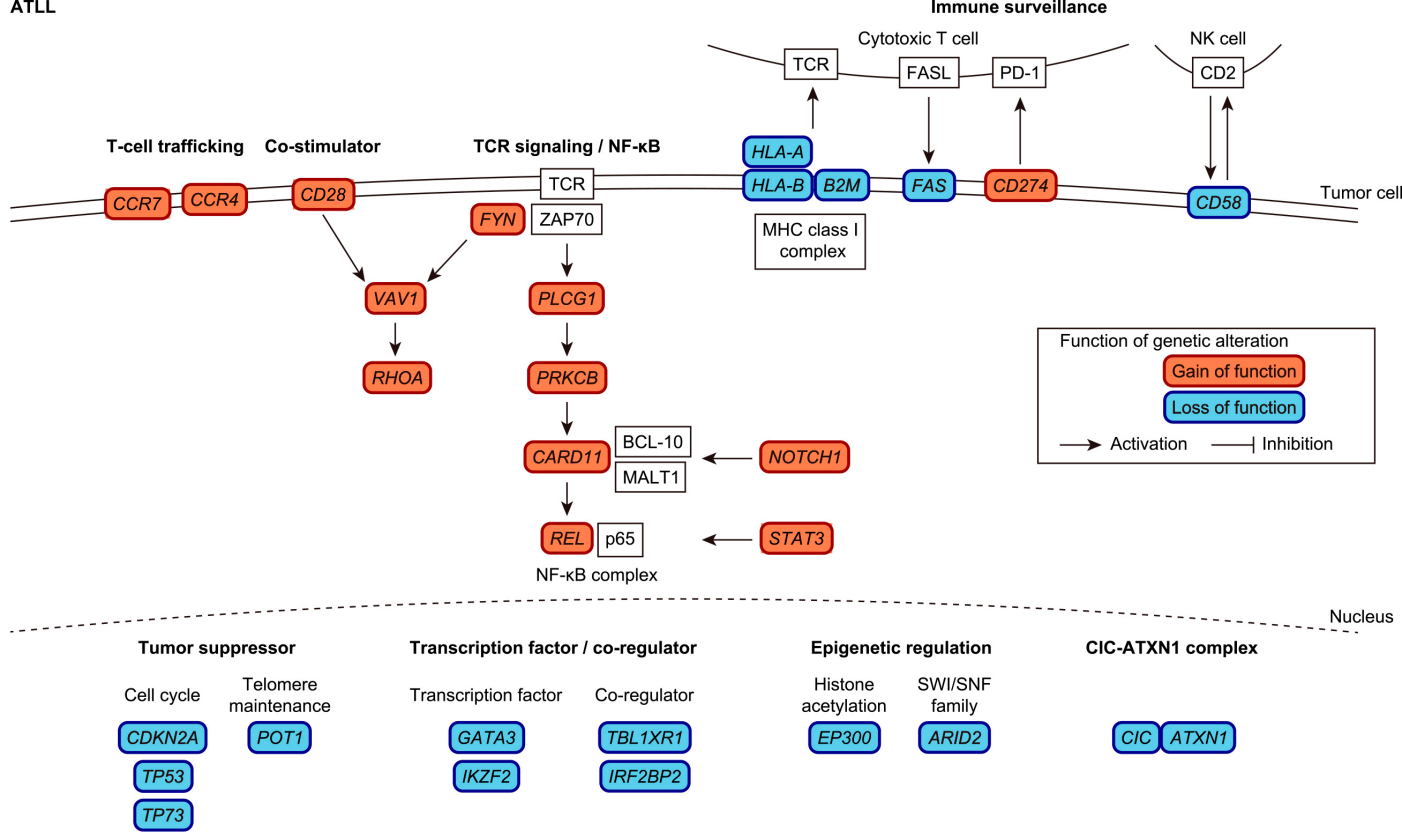
Frequent genetic alterations in the ATLL are summarized according to their functionalities. Gain-of-function and loss-of-function alterations are shown in orange and blue, respectively.

ATLL is an aggressive T-cell neoplasm which is caused by human T-cell leukemia virus type-1 (HTLV-1) retrovirus and associated with a poor prognosis ^(56), (57)^. Although HTLV-1-encoded products, such as Tax and HBZ, contribute to ATL development and/or progression, additional genetic alterations accumulate even in the HTLV-1 carrier state and are essential for ATLL leukemogenesis ^(58), (59), (60), (61)^. The ATLL genome is characterized by frequent alterations in the TCR/NF-κB signaling pathway, including gain-of-function mutations in *PLCG1*, *PRKCB*, and *CARD11*, *CTLA4*::*CD28* and *ICOS*::*CD28* fusions, and *REL* truncation (Figure 3) ^(62), (63), (64)^. Although Tax strongly activates the NF-kB signaling pathway, HTLV-1 sense strand genes, including Tax, are frequently downregulated or genetically silenced in ATLL ^(58), (59), (62)^. Thus, it is reasonable to speculate that ATLL cells develop alternative oncogenic mechanisms by acquiring somatic alterations in the TCR/NF-κB signaling pathway while escaping immune surveillance by repressing immunogenic viral genes. In addition, ATLL cells evade immune attack by acquiring somatic alterations in immune-related molecules. Like ENKTL, one-fourth of ATLL cases harbor SVs involving* PD-L1* 3′-UTR, which induce PD-L1 overexpression in not only tumor cells but also surrounding non-tumor cells ^(50), (65)^. In addition, loss-of-function alterations in MHC class 1 (*HLA-A*, *HLA-B* and *B2M*) and other immune-related molecules (*CD58* and *FAS*) occur in more than half of all ATLL cases ^(62), (63), (66)^. Together with genetic alterations, epigenetic repression through the extensive accumulation of DNA methylation and histone H3 lysine 27 (H3K27) trimethylation contributes to the deregulation of these driver pathways. For example, EZH2 is a component of polycomb repressive complex 2 (PRC2), which is involved in H3K27 trimethylation. Its overexpression is common in ATLL, which suppresses microRNA-31 (miR-31) and subsequently induces NIK overexpression, leading to constitutive activation of the noncanonical NF-κB pathway ^(67)^. In addition, DNA hypermethylation frequently occurs in the CpG islands of MHC class 1 genes, leading to the downregulation of these genes ^(62)^.

Another important genetic target is the CIC-ATXN1 transcriptional repressor complex, whose gain-of-function occurs via expansion of the ATXN1 polyglutamine stretch, leading to neurodegeneration in spinocerebellar ataxia type 1. In ATLL, loss-of-function mutations and disrupted SVs frequently affect long isoform (*CIC-L*)-specific exons. In combination with the disrupted SVs of *ATXN1*, CIC-ATXN1 alterations were detected in more than half of the ATLL cases ^(63)^. Other common genetic alterations include signaling pathway molecules (including *STAT3* and *NOTCH1* mutations), transcription factors (*GATA3* mutations and *IKZF2* intragenic deletions), transcriptional co-regulators (*TBL1XR1* and *IRF2BP2* mutations), chemokine receptors (*CCR4* and *CCR7* C-terminal truncating mutations), epigenetic regulators (*ARID2* and *EP300* mutations), and tumor suppressors (*TP53* and *CDKN2A* mutations and deletions as well as *TP73* intragenic deletions) ^(62), (63), (68), (69)^.

Clinically, ATLL is classified into four subtypes: acute, lymphoma, chronic, and smoldering ^(56), (57)^ The acute and lymphoma subtypes are aggressive diseases with a poor prognosis that usually require intensive therapies, including allogeneic stem cell transplantation. The chronic and smoldering subtypes generally exhibit indolent clinical course, but many of them eventually transform into more aggressive diseases. These aggressive and indolent diseases possess distinct genetic and epigenetic properties ^(63), (70)^. The aggressive subtypes exhibit increased mutations and CNAs. In particular, *TP53* and *IRF4* mutations, *PD-L1* amplification, and *CDKN2A* deletion are more common in aggressive subtypes, whereas *STAT3* mutation is more frequent in indolent ATLL. Based on clinical factors, several prognostic indices have been developed, including ATL-PI (based on stage, performance status, age, albumin, and soluble IL-2 receptor level) and the Japan Clinical Oncology Group prognostic index (JCOG-PI; based on calcium level and performance status) ^(13), (71)^. Moreover, albumin, blood urea nitrogen, and lactate dehydrogenase levels are associated with worse prognosis in chronic ATLL ^(56), (57)^. In addition to these clinical factors, genetic alterations can predict prognosis independently of clinical factors ^(70)^. Among individual somatic alterations, *PRKCB* mutation and *PD-L1* amplification are poor prognostic indicators in aggressive ATL, whereas in indolent ATL, *IRF4* mutation, *PD-L1* amplification, and *CDKN2A* deletion independently predict worse survival ^(70)^. In addition, the profiling of various driver alterations, including coding and noncoding mutations, SVs, and CNAs, can classify ATLL cases into two molecular groups with distinct clinical and genetic features ^(63)^. In this classification, group 1 showed fewer driver alterations but was enriched with alterations affecting proximal TCR signaling molecules (including *PLCG1* and *VAV1*) and *STAT3*. Conversely, group 2 included most lymphoma cases, showed shorter survival, and was associated with distal TCR/NF-κB pathway components (*PRKCB* and *IRF4*), immune-related molecules, and epigenetic regulators. These observations suggest that ATLL can be divided into molecularly different subsets with different clinical characteristics.

Several novel agents have been used for ATLL in clinical settings, including anti-CCR4 antibody (mogamulizumab), HDAC inhibitor (chidamide), and EZH1/2 inhibitor (valemetostat). Genetic alterations are also associated with response to novel agents. For example, *CCR4* mutation predicts a better response to mogamulizumab ^(72)^. In contrast, loss-of-function mutations and deletions of *CCR4* were identified as mogamulizumab resistance mechanisms in a subset of CTCL patients ^(73)^. Similarly, acquired mutations at the PRC2-compound interface reduced interactions with valemetostat, leading to increased H3K27 trimethylation and therapeutic resistance ^(74)^. Therefore, molecular profiling can not only improve prognostic stratification and help predict the efficacy of molecularly targeted agents in ATLL.

## Conclusion

Next-generation sequencing technologies have clarified the entire landscape of genetic alterations and identified many driver genes in various PTCL subtypes. Genetic alterations showed extensive heterogeneity within and across subtypes. In particular, several alterations are highly specific to a certain subtype, such as *RHO*A G17V and *IDH2* mutations in nTFHLs, *ALK* fusions in ALCL, *DDX3X* and *MSN* mutations in ENKTL, *PRKCB*, *CIC*, and *CCR4* mutations in ATLL. However, there are many alterations shared among different PTCL subtypes, including alterations involving TCR/NF-κB and JAK/STAT pathway molecules, epigenetic regulators, immune-associated molecules, and tumor suppressors. These genetic alterations can improve the prognostic prediction of several subtypes of PTCLs, such as ENKTL and ATLL. In addition, several genetic alterations may have the potential to predict a response to a specific molecularly targeted agent, such as *PD-L1* SVs, in response to immune checkpoint inhibitors. The genetic information provides novel insights into the molecular basis of PTCLs, which can be exploited for further diagnostic and therapeutic development to improve treatment strategies for these diseases.

## Article Information

This article is based on the study, which received the Medical Research Encouragement Prize of The Japan Medical Association in 2023.

### Conflicts of Interest

Y.K. has received honoraria from Kyowa Kirin, Nippon Shinyaku, Daiichi Sankyo, and Takeda Pharmaceutical. K.K. has received honoraria from Ono Pharmaceutical, Eisai, Astellas Pharma, Novartis, Chugai Pharmaceutical, AstraZeneca, Sumitomo Dainippon Pharma, Kyowa Kirin, Janssen Pharmaceutical, MSD, Takeda Pharmaceutical, SymBio Pharmaceuticals, Bristol-Myers Squibb, Pfizer, Nippon Shinyaku, Daiichi Sankyo, Alexion Pharmaceuticals, AbbVie, Meiji Seika Pharma, Sanofi, Sysmex, Mundipharma, Incyte Corporation, Kyorin Pharmaceutical, PharmaEssentia Japan, Gilead Sciences, Kowa, Amgen, Nippon Kayaku, and Asahi Kasei Pharma; has received research funding from Asahi Kasei Pharma, Eisai, Otsuka Pharmaceutical, Kyowa Kirin, Shionogi, Daiichi Sankyo, Takeda Pharmaceutical, Sumitomo Dainippon Pharma, Chugai Pharmaceutical, Teijin Pharma, Japan Blood Products Organization, Mochida Pharmaceutical, JCR Pharmaceuticals, Nippon Shinyaku, Nippon Kayaku, Chordia Therapeutics, Genmab, Bristol-Myers Squibb, Meiji Seika Pharma, and Regeneron; has received research funding from Meiji Seika Pharma and Otsuka Pharmaceutical; holds individual stocks in Asahi Genomics; and has a patent for genetic alterations as a biomarker in T-cell lymphomas licensed to Kyoto University and PD-L1 abnormalities as a predictive biomarker for immune checkpoint blockade therapy licensed to Kyoto University.

### Sources of Funding

This work was supported by the Japan Society for the Promotion of Science (JSPS) KAKENHI (JP21H05051), the Japan Science and Technology Agency Moonshot R&D Program (JPMJMS2022), and the Takeda Science Foundation.

## References

[1] Alaggio R, Amador C, Anagnostopoulos I, et al. The 5th edition of the World Health Organization classification of haematolymphoid tumours: lymphoid neoplasms. Leukemia. 2022;36(7):1720-48. 35732829 10.1038/s41375-022-01620-2 PMC9214472

[2] Campo E, Jaffe ES, Cook JR, et al. The international consensus classification of mature lymphoid neoplasms: a report from the clinical advisory committee. Blood. 2022;140(11):1229-53. 35653592 10.1182/blood.2022015851 PMC9479027

[3] Ngu HS, Savage KJ. Frontline management of nodal peripheral T-cell lymphomas. Am Soc Clin Oncol Educ Book. 2023;43:e390334. 37262395 10.1200/EDBK_390334

[4] Horwitz S, O'Connor OA, Pro B, et al. Brentuximab vedotin with chemotherapy for CD30-positive peripheral T-cell lymphoma (ECHELON-2): a global, double-blind, randomised, phase 3 trial. Lancet. 2019;393(10168):229-40. 30522922 10.1016/S0140-6736(18)32984-2 PMC6436818

[5] Chiba S, Sakata-Yanagimoto M. Advances in understanding of angioimmunoblastic T-cell lymphoma. Leukemia. 2020;34(10):2592-606. 32704161 10.1038/s41375-020-0990-y PMC7376827

[6] Vose J, Armitage J, Weisenburger D, et al. International peripheral T-cell and natural killer/T-cell lymphoma study: pathology findings and clinical outcomes. J Clin Oncol. 2008;26(25):4124-30. 18626005 10.1200/JCO.2008.16.4558

[7] Ellin F, Landström J, Jerkeman M, et al. Real-world data on prognostic factors and treatment in peripheral T-cell lymphomas: a study from the Swedish Lymphoma Registry. Blood. 2014;124(10):1570-7. 25006130 10.1182/blood-2014-04-573089

[8] Gallamini A, Stelitano C, Calvi R, et al. Peripheral T-cell lymphoma unspecified (PTCL-U): a new prognostic model from a retrospective multicentric clinical study. Blood. 2004;103(7):2474-9. 14645001 10.1182/blood-2003-09-3080

[9] Weisenburger DD, Savage KJ, Harris NL, et al. Peripheral T-cell lymphoma, not otherwise specified: a report of 340 cases from the International Peripheral T-Cell Lymphoma Project. Blood. 2011;117(12):3402-8. 21270441 10.1182/blood-2010-09-310342

[10] Went P, Agostinelli C, Gallamini A, et al. Marker expression in peripheral T-cell lymphoma: a proposed clinical-pathologic prognostic score. J Clin Oncol. 2006;24(16):2472-9. 16636342 10.1200/JCO.2005.03.6327

[11] Federico M, Rudiger T, Bellei M, et al. Clinicopathologic characteristics of angioimmunoblastic T-cell lymphoma: analysis of the international peripheral T-cell lymphoma project. J Clin Oncol. 2013;31(2):240-6. 22869878 10.1200/JCO.2011.37.3647 PMC3532394

[12] Advani RH, Skrypets T, Civallero M, et al. Outcomes and prognostic factors in angioimmunoblastic T-cell lymphoma: final report from the international T-cell project. Blood. 2021;138(3):213-20. 34292324 10.1182/blood.2020010387 PMC8493974

[13] Katsuya H, Yamanaka T, Ishitsuka K, et al. Prognostic index for acute- and lymphoma-type adult T-cell leukemia/lymphoma. J Clin Oncol. 2012;30(14):1635-40. 22473153 10.1200/JCO.2011.38.2101

[14] Kim SJ, Yoon DH, Jaccard A, et al. A prognostic index for natural killer cell lymphoma after non-anthracycline-based treatment: a multicentre, retrospective analysis. Lancet Oncol. 2016;17(3):389-400. 26873565 10.1016/S1470-2045(15)00533-1

[15] Couronné L, Bastard C, Bernard OA. TET2 and DNMT3A mutations in human T-cell lymphoma. N Engl J Med. 2012;366(1):95-6. 22216861 10.1056/NEJMc1111708

[16] Fujisawa M, Nguyen TB, Abe Y, et al. Clonal germinal center B cells function as a niche for T-cell lymphoma. Blood. 2022;140(18):1937-50. 35921527 10.1182/blood.2022015451 PMC10653021

[17] Palomero T, Couronné L, Khiabanian H, et al. Recurrent mutations in epigenetic regulators, RHOA and FYN kinase in peripheral T cell lymphomas. Nat Genet. 2014;46(2):166-70. 24413734 10.1038/ng.2873 PMC3963408

[18] Sakata-Yanagimoto M, Enami T, Yoshida K, et al. Somatic RHOA mutation in angioimmunoblastic T cell lymphoma. Nat Genet. 2014;46(2):171-5. 24413737 10.1038/ng.2872

[19] Yoo HY, Sung MK, Lee SH, et al. A recurrent inactivating mutation in RHOA GTPase in angioimmunoblastic T cell lymphoma. Nat Genet. 2014;46(4):371-5. 24584070 10.1038/ng.2916

[20] Cairns RA, Iqbal J, Lemonnier F, et al. IDH2 mutations are frequent in angioimmunoblastic T-cell lymphoma. Blood. 2012;119(8):1901-3. 22215888 10.1182/blood-2011-11-391748 PMC3293643

[21] Cortes JR, Ambesi-Impiombato A, Couronné L, et al. RHOA G17V induces T follicular helper cell specification and promotes lymphomagenesis. Cancer Cell. 2018;33(2):259-73.e7. 29398449 10.1016/j.ccell.2018.01.001 PMC5811310

[22] Leca J, Lemonnier F, Meydan C, et al. IDH2 and TET2 mutations synergize to modulate T Follicular Helper cell functional interaction with the AITL microenvironment. Cancer Cell. 2023;41(2):323-39.e10. 36736318 10.1016/j.ccell.2023.01.003

[23] Huang Y-H, Qiu Y-R, Zhang Q-L, et al. Genomic and transcriptomic profiling of peripheral T cell lymphoma reveals distinct molecular and microenvironment subtypes. Cell Rep Med. 2024;5(2):101416. 38350451 10.1016/j.xcrm.2024.101416 PMC10897627

[24] Vallois D, Dobay MPD, Morin RD, et al. Activating mutations in genes related to TCR signaling in angioimmunoblastic and other follicular helper T-cell-derived lymphomas. Blood. 2016;128(11):1490-502. 27369867 10.1182/blood-2016-02-698977

[25] Vallois D, Dupuy A, Lemonnier F, et al. RNA fusions involving CD28 are rare in peripheral T-cell lymphomas and concentrate mainly in those derived from follicular helper T cells. Haematologica. 2018;103(8):e360-3. 29545337 10.3324/haematol.2017.186767 PMC6068042

[26] Odejide O, Weigert O, Lane AA, et al. A targeted mutational landscape of angioimmunoblastic T-cell lymphoma. Blood. 2014;123(9):1293-6. 24345752 10.1182/blood-2013-10-531509 PMC4260974

[27] Falchi L, Ma H, Klein S, et al. Combined oral 5-azacytidine and Romidepsin are highly effective in patients with PTCL: a multicenter phase 2 study. Blood. 2021;137(16):2161-70. 33171487 10.1182/blood.2020009004

[28] O'Connor OA, Horwitz S, Masszi T, et al. Belinostat in patients with relapsed or refractory peripheral T-cell lymphoma: results of the pivotal Phase II BELIEF (CLN-19) study. J Clin Oncol. 2015;33(23):2492-9. 26101246 10.1200/JCO.2014.59.2782 PMC5087312

[29] Pro B, Horwitz SM, Prince HM, et al. Romidepsin induces durable responses in patients with relapsed or refractory angioimmunoblastic T-cell lymphoma. Hematol Oncol. 2017;35(4):914-7. 27402335 10.1002/hon.2320 PMC5763404

[30] Shi Y, Dong M, Hong X, et al. Results from a multicenter, open-label, pivotal phase II study of chidamide in relapsed or refractory peripheral T-cell lymphoma. Ann Oncol. 2015;26(8):1766-71. 26105599 10.1093/annonc/mdv237

[31] Lemonnier F, Dupuis J, Sujobert P, et al. Treatment with 5-azacytidine induces a sustained response in patients with angioimmunoblastic T-cell lymphoma. Blood. 2018;132(21):2305-9. 30279227 10.1182/blood-2018-04-840538

[32] Iqbal J, Wright G, Wang C, et al. Gene expression signatures delineate biological and prognostic subgroups in peripheral T-cell lymphoma. Blood. 2014;123(19):2915-23. 24632715 10.1182/blood-2013-11-536359 PMC4014836

[33] Watatani Y, Sato Y, Miyoshi H, et al. Molecular heterogeneity in peripheral T-cell lymphoma, not otherwise specified revealed by comprehensive genetic profiling. Leukemia. 2019;33(12):2867-83. 31092896 10.1038/s41375-019-0473-1

[34] Heavican TB, Bouska A, Yu J, et al. Genetic drivers of oncogenic pathways in molecular subgroups of peripheral T-cell lymphoma. Blood. 2019;133(15):1664-76. 30782609 10.1182/blood-2018-09-872549 PMC6460420

[35] Johnson WT, Ganesan N, Epstein-Peterson ZD, et al. TP53 mutations identify high-risk events for peripheral T-cell lymphoma treated with CHOP-based chemotherapy. Blood Adv. 2023;7(17):5172-86. 37078708 10.1182/bloodadvances.2023009953 PMC10480533

[36] Herek TA, Bouska A, Lone W, et al. DNMT3A mutations define a unique biological and prognostic subgroup associated with cytotoxic T cells in PTCL-NOS. Blood. 2022;140(11):1278-90. 35639959 10.1182/blood.2021015019 PMC9479030

[37] Mossé YP, Voss SD, Lim MS, et al. Targeting ALK with crizotinib in pediatric anaplastic large cell lymphoma and inflammatory myofibroblastic tumor: a Children's Oncology Group Study. J Clin Oncol. 2017;35(28):3215-21. 28787259 10.1200/JCO.2017.73.4830 PMC5617123

[38] Crescenzo R, Abate F, Lasorsa E, et al. Convergent mutations and kinase fusions lead to oncogenic STAT3 activation in anaplastic large cell lymphoma. Cancer Cell. 2015;27(4):516-32. 25873174 10.1016/j.ccell.2015.03.006 PMC5898430

[39] Parrilla Castellar ER, Jaffe ES, Said JW, et al. ALK-negative anaplastic large cell lymphoma is a genetically heterogeneous disease with widely disparate clinical outcomes. Blood. 2014;124(9):1473-80. 24894770 10.1182/blood-2014-04-571091 PMC4148769

[40] Pedersen MB, Hamilton-Dutoit SJ, Bendix K, et al. DUSP22 and TP63 rearrangements predict outcome of ALK-negative anaplastic large cell lymphoma: a Danish cohort study. Blood. 2017;130(4):554-7. 28522440 10.1182/blood-2016-12-755496 PMC5533203

[41] Wang H, Fu B-B, Gale RP, et al. NK-/T-cell lymphomas. Leukemia. 2021;35(9):2460-8. 34117356 10.1038/s41375-021-01313-2 PMC8410593

[42] Tse E, Zhao W-L, Xiong J, et al. How we treat NK/T-cell lymphomas. J Hematol Oncol. 2022;15(1):74. 35659326 10.1186/s13045-022-01293-5 PMC9164389

[43] Damania B, Kenney SC, Raab-Traub N. Epstein-Barr virus: biology and clinical disease. Cell. 2022;185(20):3652-70. 36113467 10.1016/j.cell.2022.08.026 PMC9529843

[44] Küçük C, Jiang B, Hu X, et al. Activating mutations of STAT5B and STAT3 in lymphomas derived from γδ-T or NK cells. Nat Commun. 2015;6(1):6025. 25586472 10.1038/ncomms7025 PMC7743911

[45] Jiang L, Gu ZH, Yan ZX, et al. Exome sequencing identifies somatic mutations of DDX3X in natural killer/T-cell lymphoma. Nat Genet. 2015;47(9):1061-6. 26192917 10.1038/ng.3358

[46] Dobashi A, Tsuyama N, Asaka R, et al. Frequent BCOR aberrations in extranodal NK/T-Cell lymphoma, nasal type. Genes Chromosomes Cancer. 2016;55(5):460-71. 26773734 10.1002/gcc.22348

[47] Xiong J, Cui BW, Wang N, et al. Genomic and transcriptomic characterization of natural killer T cell lymphoma. Cancer Cell. 2020;37(3):403-19.e6. 32183952 10.1016/j.ccell.2020.02.005

[48] Ito Y, Marouf A, Kogure Y, et al. Comprehensive genetic profiling reveals frequent alterations of driver genes on the X chromosome in extranodal NK/T-cell lymphoma. Cancer Res. 2024;84(13):2181-201. 38657099 10.1158/0008-5472.CAN-24-0132

[49] Kataoka K, Miyoshi H, Sakata S, et al. Frequent structural variations involving programmed death ligands in Epstein-Barr virus-associated lymphomas. Leukemia. 2019;33(7):1687-99. 30683910 10.1038/s41375-019-0380-5 PMC6755969

[50] Kataoka K, Shiraishi Y, Takeda Y, et al. Aberrant PD-L1 expression through 3'-UTR disruption in multiple cancers. Nature. 2016;534(7607):402-6. 27281199 10.1038/nature18294

[51] Okuno Y, Murata T, Sato Y, et al. Defective Epstein-Barr virus in chronic active infection and haematological malignancy. Nat Microbiol. 2019;4(3):404-13. 30664667 10.1038/s41564-018-0334-0

[52] Peng RJ, Han BW, Cai QQ, et al. Genomic and transcriptomic landscapes of Epstein-Barr virus in extranodal natural killer T-cell lymphoma. Leukemia. 2019;33(6):1451-62. 30546078 10.1038/s41375-018-0324-5 PMC6756073

[53] Jung YJ, Choi H, Kim H, et al. MicroRNA miR-BART20-5p stabilizes Epstein-Barr virus latency by directly targeting BZLF1 and BRLF1. J Virol. 2014;88(16):9027-37. 24899173 10.1128/JVI.00721-14 PMC4136301

[54] Yates JL, Camiolo SM, Bashaw JM. The minimal replicator of Epstein-Barr virus oriP. J Virol. 2000;74(10):4512-22. 10775587 10.1128/jvi.74.10.4512-4522.2000 PMC111971

[55] Lim JQ, Huang D, Tang T, et al. Whole-genome sequencing identifies responders to Pembrolizumab in relapse/refractory natural-killer/T cell lymphoma. Leukemia. 2020;34(12):3413-9. 32753688 10.1038/s41375-020-1000-0 PMC7685978

[56] Ishitsuka K, Tamura K. Human T-cell leukaemia virus type I and adult T-cell leukaemia-lymphoma. Lancet Oncol. 2014;15(11):e517-26. 25281470 10.1016/S1470-2045(14)70202-5

[57] Cook LB, Fuji S, Hermine O, et al. Revised adult T-cell leukemia-lymphoma international consensus meeting report. J Clin Oncol. 2019;37(8):677-87. 30657736 10.1200/JCO.18.00501 PMC6494249

[58] Matsuoka M, Jeang KT. Human T-cell leukaemia virus type 1 (HTLV-1) infectivity and cellular transformation. Nat Rev Cancer. 2007;7(4):270-80. 17384582 10.1038/nrc2111

[59] Kogure Y, Kataoka K. Genetic alterations in adult T-cell leukemia/lymphoma. Cancer Sci. 2017;108(9):1719-25. 28627735 10.1111/cas.13303 PMC5581529

[60] Yamagishi M, Kubokawa M, Kuze Y, et al. Chronological genome and single-cell transcriptome integration characterizes the evolutionary process of adult T cell leukemia-lymphoma. Nat Commun. 2021;12(1):4821. 34376672 10.1038/s41467-021-25101-9 PMC8355240

[61] Rowan AG, Dillon R, Witkover A, et al. Evolution of retrovirus-infected premalignant T-cell clones prior to adult T-cell leukemia/lymphoma diagnosis. Blood. 2020;135(23):2023-32. 32160278 10.1182/blood.2019002665 PMC7381760

[62] Kataoka K, Nagata Y, Kitanaka A, et al. Integrated molecular analysis of adult T cell leukemia/lymphoma. Nat Genet. 2015;47(11):1304-15. 26437031 10.1038/ng.3415

[63] Kogure Y, Kameda T, Koya J, et al. Whole-genome landscape of adult T-cell leukemia/lymphoma. Blood. 2022;139(7):967-82. 34695199 10.1182/blood.2021013568 PMC8854674

[64] Yoshida N, Shigemori K, Donaldson N, et al. Genomic landscape of young ATLL patients identifies frequent targetable CD28 fusions. Blood. 2020;135(17):1467-71. 31961925 10.1182/blood.2019001815 PMC7180081

[65] Koya J, Saito Y, Kameda T, et al. Single-cell analysis of the multicellular ecosystem in viral carcinogenesis by HTLV-1. Blood Cancer Discov. 2021;2(5):450-67. 34661162 10.1158/2643-3230.BCD-21-0044 PMC8514013

[66] Tamaki K, Morishima S, Suzuki S, et al. Full-length HLA sequencing in adult T cell leukemia-lymphoma uncovers multiple gene alterations. Leukemia. 2021;35(10):2998-3001. 34518643 10.1038/s41375-021-01403-1 PMC8478651

[67] Yamagishi M, Nakano K, Miyake A, et al. Polycomb-mediated loss of miR-31 activates NIK-dependent NF-κB pathway in adult T cell leukemia and other cancers. Cancer Cell. 2012;21(1):121-35. 22264793 10.1016/j.ccr.2011.12.015

[68] Nakagawa M, Schmitz R, Xiao W, et al. Gain-of-function CCR4 mutations in adult T cell leukemia/lymphoma. J Exp Med. 2014;211(13):2497-505. 25488980 10.1084/jem.20140987 PMC4267233

[69] Ong JZL, Yokomori R, Wong RWJ, et al. Requirement for TP73 and genetic alterations originating from its intragenic super-enhancer in adult T-cell leukemia. Leukemia. 2022;36(9):2293-305. 35908104 10.1038/s41375-022-01655-5

[70] Kataoka K, Iwanaga M, Yasunaga J-i, et al. Prognostic relevance of integrated genetic profiling in adult T-cell leukemia/lymphoma. Blood. 2018;131(2):215-25. 29084771 10.1182/blood-2017-01-761874 PMC5757690

[71] Fukushima T, Nomura S, Shimoyama M, et al. Japan Clinical Oncology Group (JCOG) prognostic index and characterization of long-term survivors of aggressive adult T-cell leukaemia-lymphoma (JCOG0902A). Br J Haematol. 2014;166(5):739-48. 24931507 10.1111/bjh.12962

[72] Sakamoto Y, Ishida T, Masaki A, et al. CCR4 mutations associated with superior outcome of adult T-cell leukemia/lymphoma under mogamulizumab treatment. Blood. 2018;132(7):758-61. 29930010 10.1182/blood-2018-02-835991

[73] Beygi S, Duran GE, Fernandez-Pol S, et al. Resistance to mogamulizumab is associated with loss of CCR4 in cutaneous T-cell lymphoma. Blood. 2022;139(26):3732-6. 35436328 10.1182/blood.2021014468 PMC9247360

[74] Yamagishi M, Kuze Y, Kobayashi S, et al. Mechanisms of action and resistance in histone methylation-targeted therapy. Nature. 2024;627(8002):221-8. 38383791 10.1038/s41586-024-07103-x PMC10917674

